# Improvement of sleep quality in isolated metastatic patients with spinal cord compression after surgery

**DOI:** 10.1186/s12957-023-02895-0

**Published:** 2023-01-17

**Authors:** Shuang Cao, Baoquan Xin, Yue Yu, Cheng Peng, Chengzhang Zhu, Mengqiu Deng, Xin Gao, Jianjun Chu, Tielong Liu

**Affiliations:** 1 Department of Orthopedics, Changzheng Hospital, Naval Medical University, Shanghai, China; 2 School of Health Science and Engineering, University of Shanghai for Science and Technology, Shanghai, China; 3 Department of Anesthesiology, Chaohu Hospital of Anhui Medical University, Hefei, Anhui Province China; 4 Department of Anesthesiology, Changzheng Hospital, Naval Medical University, Shanghai, China; 5 Department of Orthopedics, the Second People’s Hospital of Hefei, Hefei, Anhui Province, China

**Keywords:** Quality of sleep, Isolated spinal metastasis, Metastatic spinal cord compression, Total en bloc spondylectomy, Palliative surgery with stereotactic radiosurgery

## Abstract

**Background:**

This study aimed to assess changes in quality of sleep (QoS) in isolated metastatic patients with spinal cord compression following two different surgical treatments and identify potential contributing factors associated with QoS improvement.

**Methods:**

We reviewed 49 patients with isolated spinal metastasis at our spinal tumor center between December 2017 and May 2021. Total en bloc spondylectomy (TES) and palliative surgery with postoperative stereotactic radiosurgery (PSRS) were performed on 26 and 23 patients, respectively. We employed univariate and multivariate analyses to identify the potential prognostic factors affecting QoS.

**Results:**

The total Pittsburgh Sleep Quality Index (PSQI) score improved significantly 6 months after surgery. Univariate analysis indicated that age, pain worsening at night, decrease in visual analog scale (VAS), increase in Eastern Cooperative Oncology Group performance score (ECOG-PS), artificial implant in focus, and decrease in epidural spinal cord compression (ESCC) scale values were potential contributing factors for QoS. Multivariate analysis indicated that the ESCC scale score decreased as an independent prognostic factor.

**Conclusions:**

Patients with spinal cord compression caused by the metastatic disease had significantly improved QoS after TES and PSRS treatment. Moreover, a decrease in ESCC scale value of > 1 was identified as a favorable contributing factor associated with PSQI improvement. In addition, TES and PSRS can prevent recurrence by achieving efficient local tumor control to improve indirect sleep. Accordingly, timely and effective surgical decompression and recurrence control are critical for improving sleep quality.

## Introduction

Spinal metastases are a progressive, more prevalent, and intractable disease, with approximately 60% of osseous metastatic cases and an estimated 350,000 people dying annually from bone metastases in the USA [1–3]. Spinal metastases can cause pain, functional impairment, and worsening performance status, depending on the extent of spinal cord compression [1, 4]. Once metastatic epidural spinal cord compression (MSCC) occurs, symptoms of severe pain, sensory and motor deficits, visceral dysfunction, and loss of ambulation appear, and quality of life (QoL) can be seriously affected [5]. To prevent disease deterioration, surgical treatments such as TES and PSRS are available to restore the physiological status and improve QoL [1, 6, 7].

Sleep is critical to human well-being, learning, memory, and energy improvement. Furthermore, sleep disturbance is linked to numerous illnesses, including heart disease, obesity, diabetes, depression, and even cancer [8–10]. In the cancer population, the incidence rate of sleep disturbance is 17–70% [11], higher than 9–27% in the general population [12]. Moreover, cancer itself and cancer treatments were the factors contributing to sleep disturbance, and poor sleep is associated with pain, depression, and limited mobility [13, 14]. As a result, there is no doubt that spinal tumors are strongly correlated with sleep problems. Moreover, improving sleep quality is critical for patient recovery and may indirectly increase the overall survival time [13, 15–17].

However, to our knowledge, few studies have examined QoS in patients with solitary spinal metastasis. Additionally, the effects of surgical and multidisciplinary interventions on sleep quality in these patients are poorly understood. Therefore, to improve the perioperative management of patients, this study aimed to assess changes in QoS in isolated metastatic patients with MSCC following surgical treatment (with or without adjuvant therapy) and identify potential contributing factors for improving QoS in this intractable disease.

## Materials and methods

From December 2017 to May 2021, 257 consecutive patients were diagnosed with spinal metastases. Generally, spinal surgery indications include unendurable pain, spinal instability, progressive neurologic deficits, or their combinations.

**Fig. 1:**
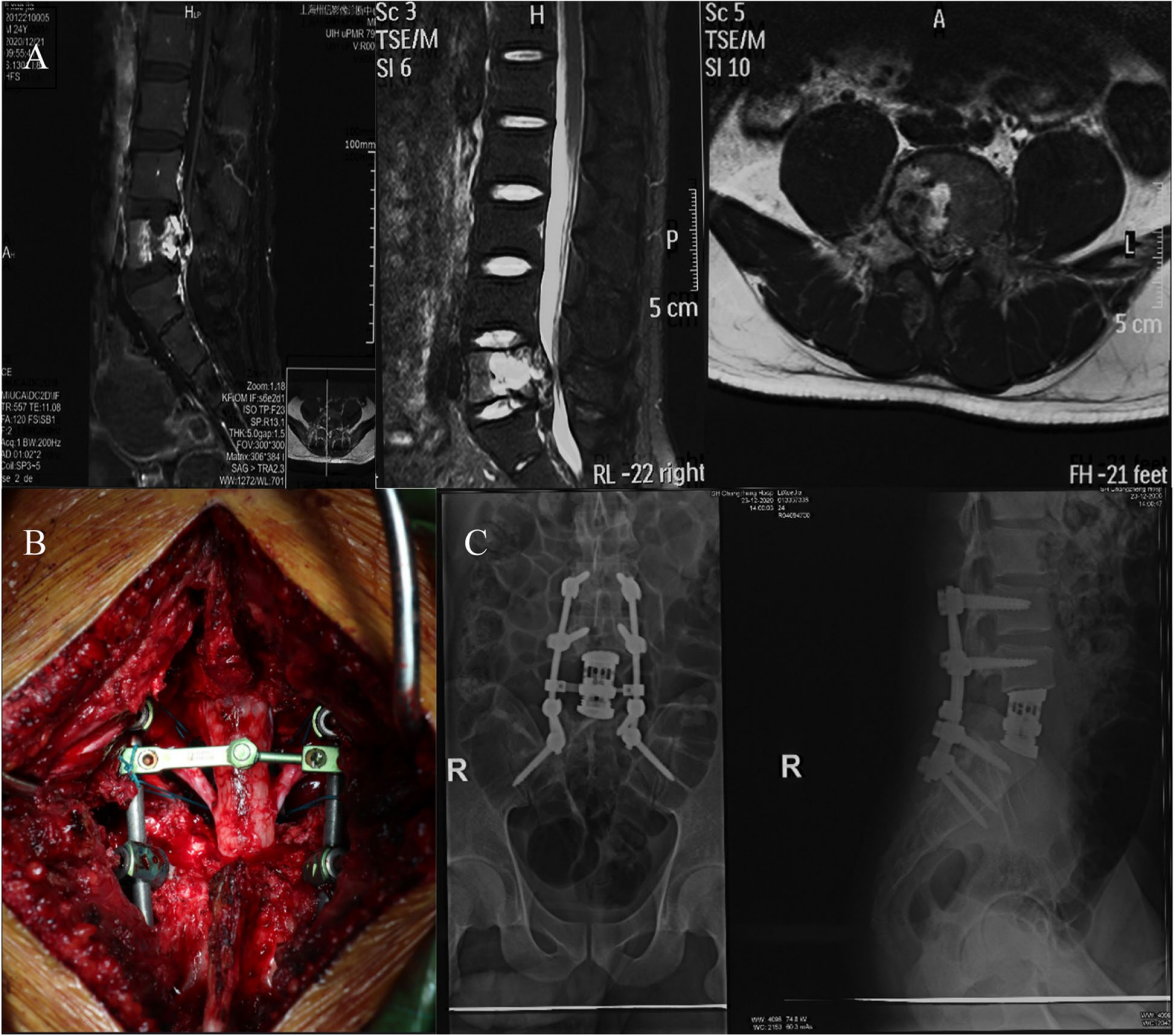
Images of a 24-year-old man with L5 metastasis from liver cancer who received TES. **A** T1-, contrast-enhanced T2-weighted and T2-MR images show the vertebral tumor extending into the spinal canal with dural sac compression. **B** Intraoperative photographs show the TES performed. **C** Postoperative X-ray

**Fig. 2:**
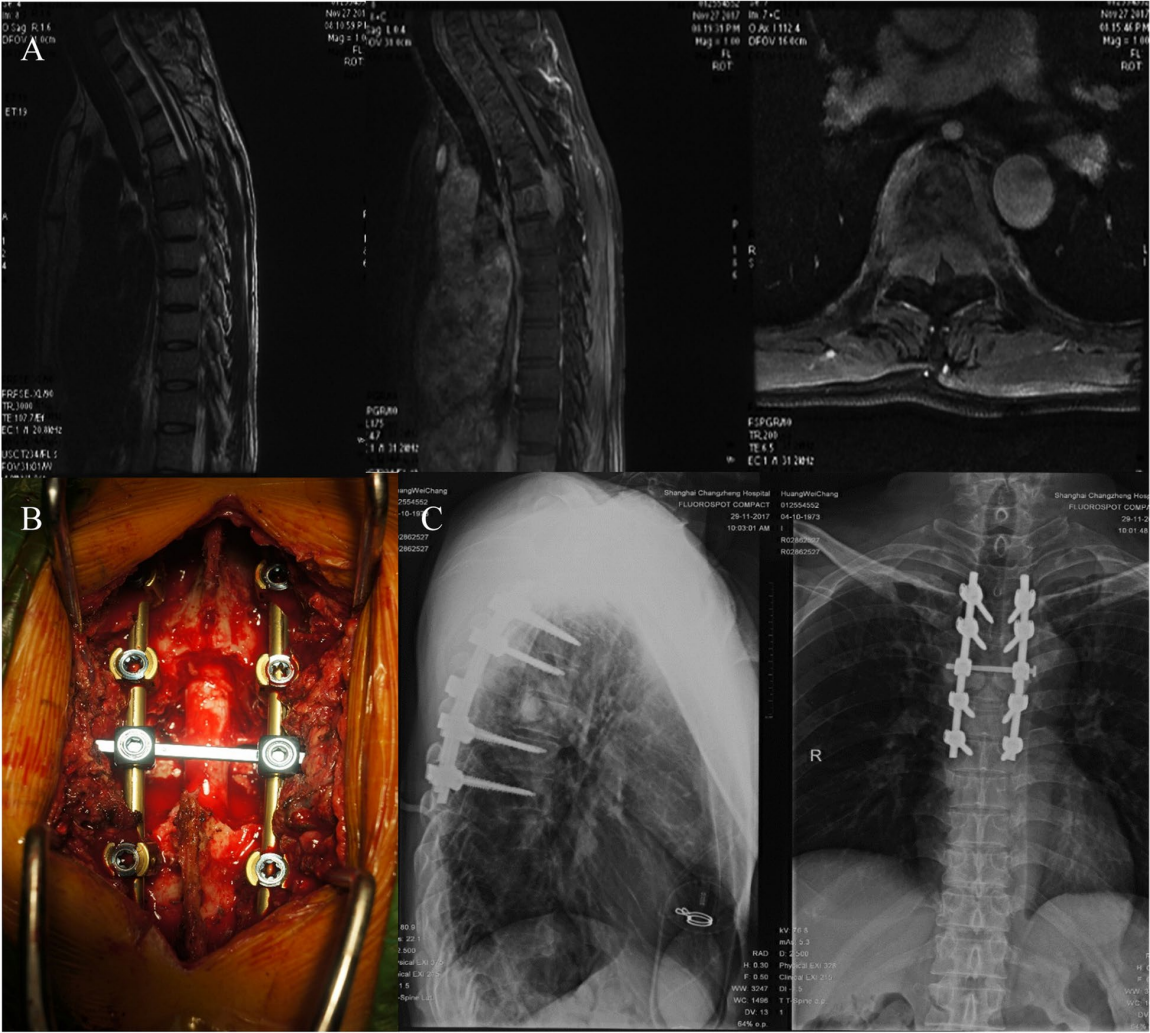
Images of a 44-year-old man with T5 metastasis from esophageal cancer who underwent palliative surgery. **A** MR images showing the vertebral tumor extending into the spinal canal with dural sac compression. **B** Intraoperative photographs displaying the PSRS performed. **C** Postoperative X-ray

Among the 257 patients, 49 were selected according to the following inclusion criteria: (1) fulfillment of the operation indication; (2) solitary spinal metastasis; (3) metastatic carcinoma in the cervical, thoracic, and lumbar spine; (4) underwent TES (Fig. 1) or PSRS (Fig. 2) operation methods; and (5) life expectancy of more than 6 months. The exclusion criteria were as follows: (1) diagnosis of psychosis or sleep apnea; (2) dyspnea interfering with sleep; (3) taking sleeping pills; (4) depression; (5) other bone metastases; (6) a history of spinal surgery; (7) other surgeries during follow-up; (8) other spinal diseases, diseases of the central nervous system, thyroid function disorder, and chronic renal or liver disease [14]; and (9) patients who were lost to follow-up. This study was approved by the ethics committee of Changzheng Hospital, and all patients or their families provided informed consent. The procedures performed in the studies involving human participants were consistent with the Helsinki Declaration of 1975, revised in 2000.

Pre- and 6-month postoperative sleep quality and patient disturbances were evaluated using a standardized local language version of the Pittsburgh Sleep Quality Index (PSQI) questionnaire [18, 19]. All questionnaires were completed in an outpatient setting. This self-report questionnaire consists of 19 items with seven subcategories: subjective sleep quality, sleep latency, sleep duration, habitual sleep efficiency, sleep disturbances, use of sleeping medication, and daytime dysfunction. Each component score ranges from 0 to 3, and the sum of the scores from all seven subcategories produces a PSQI total score ranging from 0 to 21, with higher scores indicating poorer QoS. In addition, a cutoff score of 5 indicates a threshold for sleep problems, and most poor sleepers (PSQI score > 5) were enrolled in this study. The difference in PSQI was calculated by subtracting the score at 6 months postoperative from the preoperative value. Additionally, an improvement in the global PSQI score of ≥ 3 points was considered a minimal clinically important difference [20]. In our study, improvement in PSQI ≥ 3 was classified as group A, whereas improvement in PSQI < 3 was classified as group B.

Patients who required painkillers were switched to pregabalin or gabapentin in the outpatient setting, with strict adherence to daily usage and maximum dosage, as specified in the instruction manual. Patients received postoperative intravenous patient-controlled analgesia for 24 h, followed by oral pregabalin or gabapentin, as needed, during the postoperative period. Positron emission tomography-computed tomography (PET-CT) and enhanced magnetic resonance imaging (MRI) were employed to identify the location of the lesion and the presence of another bone metastasis. The Hamilton Depression Rating Scale was used to identify depression [21]. Two independent researchers analyzed the medical records, radiographic images, and pathological reports of all 49 patients, and the procedure was conducted in a double-blind manner. The third researcher has resolved any conflicts. Body mass index (BMI), Frankel grade, Eastern Cooperative Oncology Group performance score (ECOG-PS), visual analog scale (VAS), and spinal instability neoplastic score (SINS) were employed to evaluate the degree of obesity, neurological status, performance status, degree of pain, and spinal stability, respectively. Regarding postoperative stereotactic radiosurgery in PSRS, a CT myelogram was successfully performed in all patients after surgery to delineate the spinal cord and dural margins. Within 3–4 weeks postoperatively, high-dose hypofractionated SRS was administered (dose range 24–30 Gy in 3 fractions). In addition, the planning treatment target volume was a 2–3 mm expansion of clinical tumor volume in the case of delivery uncertainties [1, 22].

Patients were followed up for a minimum of 6 months after surgery. The clinical parameters must be interpreted, including the following: (1) complications were defined as hypertension, hyperlipidemia, or diabetes diagnosed before surgery, and (2) intraoperative chemotherapy was defined as cisplatin dissolved in distilled water to soak the surgery field following tumor excision and spine reconstruction. (3) Postoperative recurrence was defined as tumors detected at the surgical site within 6 months after the operation. (4) Postoperative complications were defined as complications that required surgical or pharmacological treatment within 30 days after surgery, such as pneumonia, surgical site infection, cerebrospinal fluid leak, and internal fixation loosening. (5) Decreased epidural spinal cord compression (ESCC) scale value was used to assess the decompression degree of the spinal cord [23].

### Statistical analysis

Continuous variables were presented as mean ± SD, and differences in continuous data between groups were examined using the Student’s *t*-test. Univariate analysis was performed to compare clinical data between groups A and B and identify clinical characteristics linked to sleep quality variables. Univariate analysis included *t*-tests for continuous variables and chi-squared tests for categorical variables. Following univariate analysis, these significant variables (*P* < 0.1) were subjected to multivariate logistic regression analysis to identify significant variables affecting the PSQI scores. Statistical significance was set at *P* < 0.05. All statistical analyses were performed using SPSS, version 25.0 (IBM Corp., NY, USA).

## Results

### Patient descriptions

The preoperative mean global PSQI score was 11.80 ± 3.16, with 95.9% of patients classified as poor sleepers (PSQI score > 5). The 6-month postoperative mean global PSQI score was 5.90 ± 4.63, with only 40.8% poor sleepers. The total PSQI score decreased significantly in 43 (87.8%) of the 49 patients 6 months after surgery. Additionally, there was a statistically significant improvement between the pre- and 6-month postoperative PSQI scores (*P* < 0.001).

The baseline characteristics of the enrolled patients are presented in Table 1. There were no significant differences in age, BMI, duration of neurological symptoms, tumor size, intraoperative blood loss, and operation time between the two groups. Other baseline factors are included in Table 2, such as sex, smoking history, alcohol history, tumor site, primary tumor, surgical methods, and comorbidity. Additionally, 47.4% (*n* = 18) of the patients had comorbidities, including hypertension (*n* = 9), diabetes (*n* = 6), and hepatitis (*n* = 3) in group A. In group B, comorbidity was observed in 36.4% (*n* = 4) of patients, including hypertension (*n* = 2), diabetes (*n* = 1), and hepatitis (*n* = 1).

**Table 1:** Baseline characteristics of the enrolled patients

Factors	Group A (*n* = 38)	Group B (*n* = 11)	*P*-value
Age, years	58.45 ± 8.84 (39–72)	62.27 ± 13.21 (30–75)	0.266
BMI (kg/m^2^)	24.07 ± 2.42 (20.0–29.4)	23.06 ± 2.30 (18.20–27.50)	0.228
Duration of neurological symptoms, m	6.00 ± 7.56 (0.24–36.00)	3.30 ± 3.17 (0.25–12.00)	0.256
Tumor size, cm	4.29 ± 1.52 (1.00–6.70)	4.76 ± 1.54 (2.00–7.90)	0.369
Intraoperative blood loss, mL	1132.89 ± 696.35 (200–2000)	1072.73 ± 583.25 (200–2800)	0.795
Operation time, min	281.21 ± 85.94 (100–865)	346.82 ± 197.55 (100–504)	0.114

*P* < 0.05 is considered statistically significant

Continuous variables are presented as mean ± SD, and differences in continuous data between groups are examined using Student’s *t*-test

**Table 2:** Univariate analysis of the contributing factors affecting PSQI

Factors	*N*	△PSQI (6-month postoperative-preoperative)		
		Group A (*n* = 38)	Group B (*n* = 11)	*P*-value
**Patient-related factors**				
Sex, M/F	31/18	22/16	9/2	0.274
Age, ≤ 60 years/ > 60 years	27/22	24/14	3/8	**0.078**
BMI, ≤ 24 kg/m^2^/ > 24 kg/m^2^	29/20	22/16	7/4	> 0.999
Smoking within 2 h of bedtime, no/yes	24/25	19/19	5/6	0.791
Alcohol within 2 h of bedtime, no/yes	38/11	31/7	7/4	0.398
Duration of symptoms, ≤ 2 m/ > 2 m	22/27	16/22	6/5	0.699
Pain worsening at night, no/yes	18/31	10/28	8/3	**0.014**
Decrease in VAS, ≤ 3/ > 3	29/20	19/19	10/1	**0.037**
Increase in Frankel Grade, ≤ 2/ > 2	39/10	30/8	9/2	> 0.999
Increase in ECOG-PS, ≤ 2/ > 2	35/14	24/14	11/0	**0.045**
Comorbidity, no/yes	27/22	20/18	7/4	0.763
**Tumor-related factors**				
Tumor size, < 3 cm/ ≥ 3 cm	9/40	8/30	1/10	0.645
Tumor site, cervical/not cervical	15/34	11/27	4/7	0.921
Tumor site, thoracic/not thoracic	18/31	15/23	3/8	0.701
Tumor site, lumbar/not lumbar	16/33	12/26	4/7	> 0.999
Visceral metastasis, no/yes	28/21	20/18	8/3	0.401
Primary tumor, lung/not lung	23/26	20/18	3/8	0.138
Primary tumor, liver/not liver	7/42	5/33	2/9	> 0.999
Primary tumor, esophageal/not esophageal	1/48	0/38	1/10	0.505
Primary tumor, kidney/not kidney	3/46	1/37	2/9	0.238
Primary tumor, breast/not breast	8/41	7/31	1/10	0.784
Primary tumor, prostate/not prostate	5/44	4/34	1/10	> 0.999
Primary tumor, thyroid/not thyroid	2/47	1/37	1/10	0.930
**Treatment-related factors**				
Preoperative (2 weeks) oral analgesics (pregabalin or gabapentin), no/yes	9/40	6/32	3/8	0.672
Surgery for primary tumor, no/yes	30/19	25/13	5/6	0.386
Preoperative embolism, no/yes	37/12	28/10	9/2	0.877
Preoperative radiotherapy, no/yes	45/4	36/2	9/2	0.452
Preoperative chemotherapy, no/yes	41/8	32/6	9/2	> 0.999
Preoperative targeted therapy, no/yes	46/3	36/2	10/1	> 0.999
Operation mode, TES/PSRS	26/23	20/18	6/5	0.911
Surgical approach, posterior/combined	41/8	33/5	8/3	0.514
Internal fixed quantity, ≤ 6 / > 6	8/41	7/31	1/10	0.784
Artificial implant, bone cement/artificial vertebral body or titanium mesh	23/26	15/23	8/3	**0.052**
Intraoperative chemotherapy, no/yes	6/43	6/32	0/11	0.376
Intraoperative blood loss, ≤ 2000 mL/ > 2000 mL	45/4	34/4	11/0	0.619
Operation time, ≤ 4 h/ > 4 h	15/34	13/25	2/9	0.519
Postoperative (at 6-month follow-up) oral analgesics (pregabalin or gabapentin), no/yes	44/5	34/4	10/1	> 0.999
Postoperative radiotherapy, no/yes	23/26	18/20	5/6	0.911
Postoperative chemotherapy, no/yes	26/23	18/20	8/3	0.138
Postoperative targeted therapy, no/yes	42/7	32/6	10/1	0.944
Postoperative complications, no/yes	41/8	31/7	10/1	0.784
Spinal instability neoplastic score (SINS), ≤ 12/ > 12	41/8	32/6	9/2	> 0.999
Epidural spinal cord compression (ESCC) scale value decreased, ≤ 1/ > 1	20/29	19/19	1/10	**0.037**

*P* < 0.1 are illustrated in bold, and which is considered statistically significant in univariate analysis

The univariate analysis included *t*-tests for continuous variables and chi-square tests for categorical variables

Group A: Improvement in PSQI ≥ 3

Group B: Improvement in PSQI < 3

### Treatment

Surgical treatment was performed on all patients in our series (26 with TES and 23 with PSRS). At the 6-month follow-up, clinically meaningful improvement in sleep (a decreased global PSQI score ≥ 3 points) was observed in 76.9% (*n* = 20) of the TES treatment group and 78.3% (*n* = 18) of the PSRS treatment group. There were no differences between the two different surgical methods. The pre-, intra-, and postoperative adjuvant therapies are listed in Table 2.

There were no significant differences between the groups in oral analgesic use during the two weeks before surgery (84.2% in group A and 72.7% in group B). At the 6-month follow-up, the number of patients who required oral analgesics was significantly reduced (10.5% in group A and 9.1% in group B). however, our study did not identify differences between groups.

### Follow‑up and outcomes

The minimum follow-up duration was 6 months. In addition, recurrence was not observed in our series during the minimum follow-up period.

In group A, postoperative complications, including pneumonia (4) and weakness (3), were observed in 18.4% (*n* = 7) of the patients. The patient recovered after antibiotic therapy and symptomatic treatment. In group B, 12.5% (*n* = 1) of the patients experienced surgical site infection, and the patient underwent a second operation and recovered following debridement with antibiotic therapy. No significant differences were observed between the groups in terms of postoperative complications (*P* = 0.784).

At the 6-month follow-up, 77.4% (*n* = 24) of 31 patients with pain worsening symptoms at night before surgery were relieved operatively. Moreover, all 24 patients belonged to group A (decreased global PSQI score ≥ 3 points), and the three patients with pain worsening at night belonged to group B. In addition, pain worsening at night was significantly associated with improved QoS (*P* = 0.014) (Table 2).

All 49 operative patients experienced substantial pain relief after surgery. In group A, the mean VAS score dropped from 6.89 (range, 3–10) preoperatively to 2.96 (range, 1–6) postoperatively, and the score dropped from 6.75 (range, 4–10) to 4.36 (range, 1–7) in group B. There were significant differences between the groups in the decrease in the VAS score (*P* = 0.037).

Six months after surgery, 85.7% (*n* = 42) of patients improved by at least 1-grade on ECOG-PS, and those with a rate of 0–2 on ECOG-PS increased from 57.1% (*n* = 28) to 71.4% (*n* = 35). Moreover, all 14 patients with a higher increase in the ECOG-PS score (> 2) showed better improvement in the PSQI score (≥ 3). There was a statistically significant improvement in the PSQI between the groups when ECOG-PS > 2 (*P* = 0.045) was increased.

Regarding the ESCC scale value, 20 patients were classified as stage 1, 18 as stage 2, and 11 as stage 3. Following surgery, the compression of the spinal cord was completely relieved. Additionally, there were significant differences between the groups in terms of decreased ESCC scale scores (*P* = 0.037).

### Univariate and multivariate analyses of contributing factors

Table 2 displays the results of the univariate analysis of the contributing factors affecting sleep quality. Patients with pain symptoms worsened at night, and those using bone cement as an artificial implant in focus had a poorer improvement in PSQI (*P* = 0.014 and *P* = 0.052, respectively). Patients with a greater decrease in VAS score (> 3), greater increase in ECOG-PS (> 2), and age < 60 years had a better improvement in PSQI (*P* = 0.037, *P* = 0.045, and *P* = 0.078, respectively). In addition, a decrease in the ESCC score > 1 was an unbeneficial indicator of improvement in PSQI (*P* = 0.037). No significant differences were found in the other factors.

The above-mentioned six potential contributing factors were then subjected to multivariate logistic regression analysis (Table 3). Patients whose ESCC scale value decreased > 1 had a significantly lower risk of improvement in PSQI < 3 (*P* = 0.027) with HR of 0.025 (95% CI: 0.001–0.660), and this result as a favorable contributing factor is inconsistent with the univariate analysis one. In addition, the other five factors did not independently contribute to PSQI improvement.

**Table 3:** Multivariate analysis of contributing factors affecting PSQI

Factors	HR (95% CI)	*P*-value
Age, ≤ 60 years/ > 60 years	0.237 (0.021–2.700)	0.246
Pain worsening at night, no/yes	11.409 (0.864–150.653)	0.064
Decrease in VAS, ≤ 3/ > 3	8.678 (0.455–165.622)	0.151
Increase in ECOG-PS, ≤ 2/ > 2	-	0.998
Artificial implant in focus, bone cement / artificial vertebral body, or titanium mesh	4.326 (0.373–50.231)	0.242
Epidural spinal cord compression (ESCC) scale value decreased, ≤ 1/ > 1	0.025 (0.001–0.660)	**0.027**

*P* < 0.05 are demonstrated in bold, and which is considered statistically significant in multivariate analysis

The multivariate analysis using logistic regression analysis to identify significant variables affecting PSQI

*HR* hazard ratio,

*95% CI* 95% confidence interval

## Discussion

Sleep disorders are a common problem in patients with cancer [10], with a prevalence almost double that of the general population [24]. Patients with sleep disorders are not only more likely to suffer from clinical complications, but their general condition and physical function deteriorate as the disease progresses, impairing their ability to inhibit tumor proliferation [15, 24]. Therefore, the QoS plays a vital role in human health.

As a multidisciplinary approach has been developed for managing cancer patients, metastatic cancer has become more prevalent in clinical practice because patients live longer [1, 25, 26]. Approximately 10% of metastases occur in the spine [27]. MSCC is a severe oncological emergency that often leads to neurological sequelae and even permanent disability [1, 28]. Once MSCC occurs in patients with spinal metastases, their quality of life is extremely affected. Furthermore, sleep problems are widespread in the clinic. Good QoS can enhance overall survival by improving QoL and physical performance [13, 15–17]. Therefore, it is important to study sleep disorders in patients with spinal metastasis. To our knowledge, this study is the first to assess QoS in solitary spinal metastasis patients with MSCC treated with decompression surgery and identify potential contributing factors for QoS improvement.

According to earlier reports, adjuvant therapy can cause insomnia owing to its side effects, including headaches, nausea, night sweats, digestive symptoms, and fatigue [10]. However, adjuvant therapy (pre- or postoperative) did not affect the QoS in our study. Three reasons may account for these inconsistent conclusions. First, our study utilized timely symptomatic treatment for adjuvant therapy-related adverse events to weaken the interference of related factors. Second, the specificity of spinal metastasis may have led to different outcomes. Third, the small sample size made it difficult to draw a convincing conclusion. In addition, Nora et al. reported that higher levels of obesity are associated with poorer QoS [17]. However, similar results have not been revealed in our work, possibly due to the much higher average BMI (42.1 kg/m^2^) in their study. Although a higher BMI value could not be reached in our study due to possible ethnic or economic reasons, we still believe that the conclusion is clinically significant.

Regarding postoperative complications, a previous study suggested that sleep disorders were aggravated following its diagnosis [13]. But our study did not indicate a statistical difference, possibly because the complications were carefully solved within 6 months after surgery. However, it is noteworthy that improving the QoS in patients with surgical site infection was not evident in the patients in our study (*n* = 1). Therefore, we presume that strict aseptic operations, antibiotic therapy, and timely symptomatic treatment are essential to reduce patients, potential sleep problems. On the other hand, although Jihye et al. suggested that QoS was improved when patients used oral analgesics [29], we failed to identify obvious differences in our series. However, using oral analgesics significantly decreased at the 6-month follow-up. As a result, we suggest that painkillers are beneficial to QoS. The reason for the unparalleled conclusion may be that the follow-up interval in our study was too long to demonstrate the details of the contributing factors. Therefore, we look forward to addressing this research design gap and incorporating more extensive research into future studies.

Our univariate analysis revealed that an increase in ECOG-PS score, a decrease in VAS score, and pain worsening at night were contributing factors. Previous studies have similarly concluded that pain could impair QoS in cancer patients [13, 30, 31], ECOG-PS corresponds to neurological function, and a good general health condition in patients was able to attain a better quality of life postoperatively [15]. However, the differences between these three factors were not significant in our multivariate analysis, possibly due to confounding variables interfering with and collinearity between factors. Nevertheless, in all 14 patients with an increase in ECOG-PS score > 2, the PSQI remarkably improved (≥ 3). Consequently, we consider that intraoperative protection of nerves and rehabilitation treatment are necessary to improve QoS. Mary et al. reported similar findings, proposing that exercises (particularly aerobic exercises) are linked to QoS in cancer patients [32]. In addition, although our work did not indicate a statistically significant difference between analgesics, it is evident that pain control with painkillers can improve the general condition [13, 30].

On the one hand, MSCC is a severe oncological emergency that extremely impairs the quality of life [1]. Moreover, a previous study indicated that QoS is indirectly affected by spinal cord compression [17, 31]. As a result, timely treatment of spinal cord compression is vital for improving PSQI. In our study, univariate and multivariate analyses revealed a strong association between the PSQI and decreased ESCC scale values. Furthermore, it was interestingly found in our study that a decrease in ESCC score > 1 was an adverse contributing factor to QoS in patients with spinal metastasis in univariate analysis, while it was a favorable factor in multivariate analysis. However, it is noteworthy that among ten patients with ESCC scale value decreased by > 1 with improvement in PSQI < 3 at the 6-month follow-up; all of them increased in ECOG-PS ≤ 2, and 9 of 10 decreased in VAS ≤ 3. Therefore, we suggest that the interference of confounding factors leads to inconsistencies in univariate and multivariate analyses, and a decrease in the ESCC scale value of > 1 was a favorable factor for PSQI improvement. On the other hand, recurrence can result in anxiety and depression, which can impair the QoS of patients [17, 31]. To our knowledge, TES and PSRS operation methods could prevent recurrence by achieving efficient local tumor control [1, 16], QoS can be significantly improved following surgery, and ideal surgical findings were obtained in both operation methods in our study. Therefore, we suggest that sleep disorders can be relieved when performing timely surgery and effective recurrence control for this intractable disease.

Our study had several limitations. (1) The retrospective nature of this study is its main limitation. (2) All patients enrolled in our study underwent surgical intervention, and no control group was treated without surgery. (3) Solitary spinal metastasis, as a rare disease with strict surgical inclusion criteria, led to a small sample size in our study. (4) Due to the limited sample size, we did not design subgroups for the analyses of oral analgesics based on detailed therapeutic schemes. Therefore, the reliability of our results must be confirmed by further research, and a related study will be conducted in our subsequent work.

## Conclusions

After surgical treatment, patients with spinal cord compression caused by metastatic disease showed a significant improvement in QoS. Additionally, both TES and PSRS have been demonstrated to significantly enhance the QoS. Moreover, a decrease in the ESCC scale value > 1 was identified as a favorable contributing factor for PSQI improvement. In addition, TES and PSRS can prevent recurrence by achieving efficient local tumor control to improve indirect sleep. Accordingly, timely and effective surgical decompression and recurrence control are critical for improving sleep quality.

## Data Availability

The datasets used and/or analyzed during the current study are available from the corresponding author upon reasonable request.

## Abbreviations

QoS: Quality of sleep
TES: Total en bloc spondylectomy
PSRS: Palliative surgery with postoperative stereotactic radiosurgery
PSQI: Pittsburgh Sleep Quality Index
VAS: Visual analog scale
ECOG-PS: Eastern Cooperative Oncology Group performance score
ESCC: Epidural spinal cord compression
MSCC: Metastatic epidural spinal cord compression
QoL: Quality of life
PET-CT: Positron emission tomography-computed tomography
MRI: Magnetic resonance imaging
SINS: Spinal instability neoplastic score
BMI: Body mass index
